# Family and literature analysis demonstrates phenotypic effect of two variants in the calpain-3 gene

**DOI:** 10.1007/s10048-023-00728-6

**Published:** 2023-08-17

**Authors:** Maike Tomforde, Meike Steinbach, Tobias B. Haack, Gregor Kuhlenbäumer

**Affiliations:** 1 Department of Neurology, University Medical Center Schleswig-Holstein, Kiel, Germany; 2 Institute of Medical Genetics and Applied Genomics, University of Tübingen, Tübingen, Germany; 3 Department of Neurology, Kiel University, Arnold-Heller Str. 3, D-24105 Kiel, Germany

**Keywords:** Limb-girdle-muscular-dystrophy, Genetics, CAPN3, Genotype/phenotype

## Abstract

Both, recessive (LGMD R1) and dominant (LGMD D4) inheritance occur in calpain 3-related muscular dystrophy. We report a family with calpain-related muscular dystrophy caused by two known variants in the calpain 3 gene (*CAPN3*, NM_000070.3; (I) c.700G>A, p.Gly234Arg and (II) c.1746-20C>G, p.?). Three family members are compound heterozygous and exhibit a relatively homogeneous phenotype characterized by progressive proximal weakness starting in the third to fourth decade of life in the shoulder girdle and spreading to the legs. Two family members affected only by the p.Gly234Arg heterozygous missense variants show a different phenotype characterized by severe exertional myalgia without overt pareses. We conclude that in our family, the missense variant causes a severe myalgic phenotype without pareses that is aggravated by the second intronic variant and put these findings in the context of previous studies of the same variants.

**Supplementary Information:**

The online version contains supplementary material available at 10.1007/s10048-023-00728-6.

## Introduction

Calpain 3-related LGMD is the most common form of limb-girdle muscular dystrophy (LGMD) in most cases caused by biallelic variants in the calpain 3 gene (LGMD R1, *CAPN3*; equivalent to LGMD2A in the previous LGMD classification) [1, 2]. Heterozygous *CAPN3* variants are a less common cause of LGMD (LGMD D4; equivalent to LGMD1I in the previous LGMD classification) and are usually associated with a milder phenotype [2]. *CAPN3* variants causing LGMD were first identified in 1995 [3]. Depending on the pattern of muscle weakness, a predominantly pelvifemoral and a scapulohumeral phenotype can be distinguished [2]. The age of onset of the pelvifemoral type is more variable, ranging from childhood up to greater 30 years while the scapulohumeral type usually manifests in adults. Symmetric proximal weakness and myalgia, especially exertional myalgia, are the common symptoms. The disease is usually progressive but the rate of progression varies widely [4, 5]. Respiratory insufficiency occurs in some patients [5, 6]. Cardiomyopathy is not part of the disease. Calpains are proteases with a plethora of functions [7]. Calpain 3 is predominantly expressed in skeletal muscle and has been related to cell survival and skeletal plasticity [7]. This study aims to clarify the phenotype of calpain 3-related muscular dystrophy in a family with three individual compound-heterozygous for two variants and two individuals heterozygous for one of them.

## Case reports and genetic studies

The patients were examined in the muscle center of the Department of Neurology of Kiel University. Diagnostic genetic testing was performed by the Department of Genetics and Applied Genomics of the University of Tübingen (methods described in [8]). Written informed consent was obtained from all patients. Approval for case reports was obtained from the ethics committee of the medical faculty of Kiel University.

**Fig. 1:**
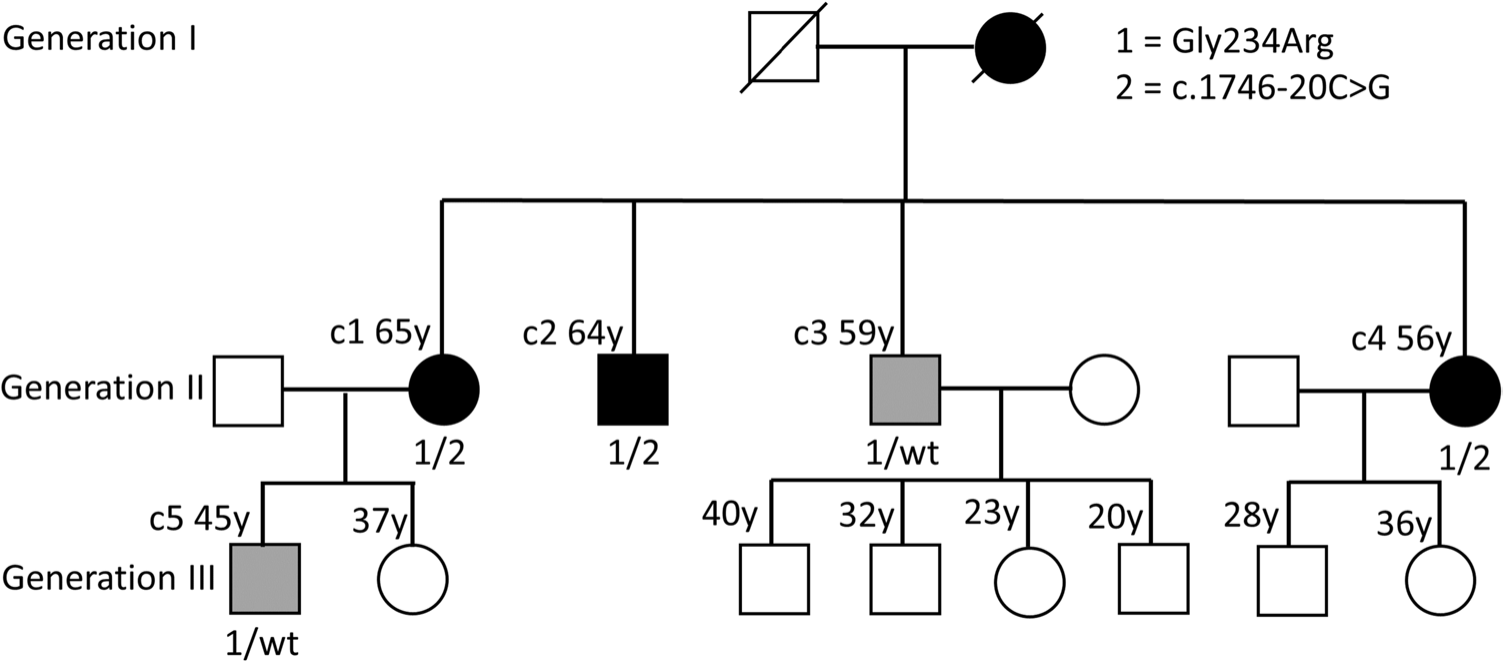
Pedigree of the family with LGMD calpain 3-related. Empty symbols—clinically unaffected individuals; symbols with black filling—clinically affected individuals carrying both, the NM_000070.3: c.700G>A, p.Gly234Arg and the NM_000070.3: c.1746-20C>G, p.? variant; symbols with gray filling—clinically affected individuals carrying the p.Gly234Arg variant only. c1 to c5—patients described in this report; XX y—current (2023) age in years; 1/2 below symbols—indicating the compound heterozygous state for both variants, 1/wt—indicating the heterozygous state for the p.Gly234Arg variant. Individuals without these symbols did not undergo molecular genetic analysis

Figure 1 shows the pedigree of the family, Table 1 the clinical characteristics, Table 2 data concerning the identified variants, and supplementary tables 1 and 2 summarize the data obtained from the literature for both variants in question. Five individuals (Fig. 1, c1 to c5) were examined clinically and genetically. All other individuals in generation III are self-reported free of muscular symptoms and did not want predictive genetic diagnostics.

**Table 1:** Summary of the clinical characteristics of patients c1 to c5

Patient	Age (years)	Sex (M—male, F—female)	Age at onset (years)	First symptom	Distribution of weakness	Myalgia	MRC UE Prox	MRC UE Dist	MRC LE Prox	MRC LE Dist	Ambulation	CK (U/l)	CAPN3
c1	65	F	~35–40	Prox arms	Arms > legs Prox > distal	No	0–3	4–5	2–3	4–5	Wheelchair, walk few steps	600	p.Gly234Arg; c.1746-20C>G
c2	64	M	~30	Prox arms	Arms > legs Prox > distal	No	3–5	5	3–4	5	Ambulant, squat not possible	900	p.Gly234Arg; c.1746-20C>G
c3	59	M	~25–30	Severe myalgia prox arms	Subjective arms	Yes, severe	5	5	5	5	Fully ambulant	70	p.Gly234Arg
c4	56	F	~30–40	Arms	Arms > legs Prox > distal	No	0–3	4–5	2–4	5	Wheelchair, walk 50 m	300, 1000	p.Gly234Arg; c.1746-20C>G
c5	45	M	40	Myalgia generalized	None	Yes, severe	5	5	5	5	Fully ambulant	137	p.Gly234Arg

*MRC UE Prox*, pareses grade according to the Medical Research Council scale in the proximal upper extremity; *MRC UE Dist*, distal upper extremity; *MRC LE Prox*, proximal lower extremity; *MRC LE Dist*, distal lower extremity

**Table 2:** Characteristics of the variants in the calpain 3 gene identified in this study

Variant	p.Gly234Arg	c.1746-20C>G
cDNA/protein level (ENST00000397163.3/NM_000070.3)	c.700G>A, p.Gly234Arg	c.1746-20C>G, p.?
Genomic level (GRCh38/hg38, NC_00015.10)	chr15:42388995G>A	chr15:42403721C>G
dbSNP	rs886042440	rs201892814
Frequency in gnomAD (v2.1.1, European, non-Finnish): (allele count/total alleles) frequency	Not present	(595/129,190) 0.004606; homozygosity rate: 0.00003 (3/100,000)
ClinVar (30.01.2023)	2 submitters, both “uncertain significance”	7 submitters, 3 “benign,” 2 “likely benign,” 2 “uncertain significance”
Leiden Open Variant Database (30.01.2023)	3 submitters, 1 “pathogenic (dominant),” 1 “likely pathogenic,” 1 “likely pathogenic (recessive)”	50 submitters, 3 “benign,” 1 “likely benign,” 14 “VUS,” 3 “likely pathogenic,” 21 “pathogenic (recessive),” 8 “pathogenic”
CADDphred (online version 30.01.2023)	25.5	6.39
PolyPhen (value/category) (via CADD website)	0.92/“probably damaging”	NA
SIFT (value/category) (via CADD website)	0/“deleterious”	NA

dbSNP—https://www.ncbi.nlm.nih.gov/projects/SNP/snp_summary.cgi; gnomAD—https://gnomad.broadinstitute.org/; ClinVar—https://www.ncbi.nlm.nih.gov/clinvar/; Leiden Open Variant Database—https://www.lovd.nl/; CADD—https://cadd.gs.washington.edu/; PolyPhen and SIFT annotations obtained via the CADD website

Patient c1, now 65 years old, reported to our clinic in 1998 (aged 41 years) with slowly progressive weakness at that time mainly affecting her shoulder girdle without involvement of facial muscles. She had been a sportive child and played handball until the age of ~35 years. A muscle biopsy showed signs of a “very discrete unspecific myopathy.” Protein and enzyme studies of the muscle biopsy not including calpain 3 were negative as well as the molecular genetic analysis for facioscapulohumeral muscular dystrophy (FSHD, 4q form). The weakness was slowly progressive and increasingly also affected the legs. The current neurologic exam showed a predominantly proximal paresis affecting the arms more than the legs (Table 1). The patient is still able to walk a few steps but is wheelchair-bound for any longer distance. Creatine kinase (CK) concentration in serum was elevated to ~600 U/l. Electromyogram (EMG) examination showed myopathic changes in the quadriceps femoris, biceps brachii, and deltoid muscles. Motor and sensory nerve conduction studies were normal. Muscle ultrasound revealed hyperechogenicity in the deltoid and biceps brachii but not in distal arm muscles or muscles of the lower extremities. Diagnostic exome analysis revealed compound heterozygosity for a missense variant (NM_000070.3: c.700G>A, p.Gly234Arg) and an intronic variant (NM_000070.3: c.1746-20C>G, p.?) in *CAPN3*. No additional rare variants of likely clinical relevance were detected in genes previously associated with the patient’s clinical features.

Patient c2, now 64 years old, trained as a carpenter and bricklayer. In his 30s, he developed progressive problems working overhead caused by proximal weakness of the arms, initially diagnosed as brachial plexus neuritis. Later additional proximal leg weakness prompted the diagnosis of myopathy. A muscle biopsy showed—as in his sister—signs of an unspecified myopathy. The patient is still ambulant but cannot get up from a squatting position. The neurologic examination showed predominantly proximal pareses, somewhat milder than in his 1-year older sister c1, and the CK concentration was elevated at around 900 U/l. Muscle biopsy, EMG, or muscle MRI was not performed. Diagnostic Sanger sequencing of the two variants found in his sister c1 showed that he carries the same variants.

Patient c3, now 59 years of age, played handball in his youth. In his 20s, he developed extremely severe myalgia during and after training eventually leading to his abandonment of the sport. Exertional myalgia progressed over the years and became so severe that he had to retire from his job as a truck driver at the age of 41 years. Currently, patient c3 also complains of proximal arm weakness but the neurologic exam did not show any paresis. The CK concentration without prior exertion was normal (~70 U/l). EMG examination showed myopathic changes mainly in the biceps brachii and deltoid muscles but mild changes in the form of polyphasic potentials also in the quadriceps femoris and anterior tibial muscles. Diagnostic Sanger sequencing of the familial variants showed that he carries only the p.Gly234Arg missense variant.

Patient c4, now 56 years old, was also a sportive girl playing handball. However, she reports that her gait has always been “a little bit waddling” and her “shoulder blades protruded.” She noticed a weakness in her arms around the age of 40 years, subsequently also affecting her legs. She is now able to walk 50 m with a walking stick but requires a wheelchair for longer distances. Neurologic examination showed predominantly proximal pareses of approximately the same severity as her 9 years older sister c1. CK concentration was elevated to ~300–1000 U/l. Muscle biopsy, EMG, or muscle MRI was not performed. Diagnostic Sanger sequencing of the familial variants showed that she carries both variants.

Patient c5, now 45 years of age, is the son of patient c1 and suffers since age ~40 from severe generalized exertional myalgia without pareses elicited even by very mild exercise. He works as a sea freight inspector, now part-time due to his disease. The neurologic examination was normal and the CK concentration without prior exertion with 137 U/l in the normal range. EMG examination of the biceps brachii and quadriceps muscles was normal. Diagnostic Sanger sequencing of the familial variants revealed heterozygosity for the p.Gly234Arg missense variant.

None of the patients exhibited so far facial weakness, speech or swallowing problems, respiratory problems, or cardiac involvement. Distal pareses were mild or absent.

## Discussion

In summary, we describe three patients with compound heterozygosity for two variants in *CAPN3* (NM_000070.3): c.700G>A, p.Gly234Arg and an intronic variant c.1746-20C>G, p.? as well as two patients carrying only the p.Gly234Arg variant in the heterozygous state. The phenotype of the compound heterozygous patients consists of predominantly proximal pareses without severe myalgia manifesting in the fourth decade subsequently leading to impaired ambulation and severe limitation of all activities requiring lifting the arms. The patients report, e.g., that they support their elbows when brushing their teeth or combing their hair. The CK concentration is elevated to ~300–1000 U/l. This phenotype has also been described as “scapulohumeral LGMD” [2]. The scapulohumeral form often shows a later onset and slower progression that the pelvifemoral form [2] which is in keeping with the late onset (25–40 years) and slow progression in our patients. Patients carrying only the p.Gly234Arg suffer from generalized myalgia upon slight exertion also manifesting in the third decade of life but without overt pareses at least up to the age of ~60 years. However, the myalgias are severe in both patients leading to early retirement in one patient and partial early retirement in the other patients. Notably, the severity of the myalgias led to molecular genetic investigations in the early 2000s which did not identify the cause of the disease.

The p.Gly234Arg variant carries all attributes of a pathogenic variant (Table 2). The variant is not found in gnomAD, the CADDphred score is high (25.5), and it is classified as most likely damaging by other tools (Table 2). To the best of our knowledge, the p.Gly234Arg variant has been described in 4 publications [9–12] but only two with scant phenotypic information (Suppl. Tab. 1 and [10, 11]). Macias et al. report a 57-year-old woman with onset in adolescence, ambulation with assistance and both *CAPN3* variants also found in our 3 compound heterozygous patients c1, c2, and c4. She had an affected deceased sister with the same *CAPN3* variants. Gonzalez-Mera et al. describe a singleton male, aged 62 with disease onset at the age of 15 years, exertional myalgia, scapular winging a waddling gait but fully ambulant. CK was increased “15-fold.” The patient was heterozygous for the p.Gly234Arg variant also carried by 2 of our patients c3 and c5 in the heterozygous state. The genotype-phenotype associations reported are therefore largely in accordance with the ones in our family, except for the fact that the onset in the compound heterozygous patient described by Macias was earlier and the heterozygous patient reported by Gonzalez-Mera was somewhat more severely affected than c3 and c5 in our family. Several other dominantly acting variants in *CAPN3* have been described and it is assumed that they exert a dominant negative effect [11, 13]. In contrast, the c.1746-20C>G variant characteristics are rather innocuous, the frequency in non-Finnish Europeans being ~0.5% and the CADDphred score 6.39 using the most recent CADD version incorporating deleterious splice-site prediction (Table 2). The variant is present in the ClinVar as well as the Leiden Open Variant Database (LOVD) with an equivocal assessment of its pathogenicity (Table 2). A recent study by Mroczek et al. focusing on c.1746-20C>G describes 14 patients and summarizes the literature concluding that c.1746-20G>C is most likely a hypomorphic variant causing LGMD in conjunction with a second pathogenic variant [14]. Through literature and database analysis Mroczek et al. also identified 7 individuals homozygous for c.1746-20G>C of whom 2 are described as affected by LGMD and 5 as not affected [14–16]. Due to its high population frequency, the c.1746-20G>C variant has been reported by at least 19 publications (Suppl. Tab. 2 and references therein). In summary, this variant was found in the heterozygous state in 18/19 publications representing 55 patients in combination with several other variants on the second allele, most commonly c.598-612del; p.Phe200_Leu204del in 16 patients. These observations are in line with two publications reporting a severe decrease in calpain 3 expression on the mRNA and protein level [17, 18] and reduced protease activity [17] as well as the formation of a new functional splice acceptor site [18] caused by c.1746-20G>C but in opposition to another study finding no difference in *CAPN3* 3 mRNA expression [19]. The prevalence of LGMD caused by *CAPN3* variants has been estimated at ~1/100,000 in non-Finnish Europeans [20]. The c.1746-20C>G variant has a homozygote frequency of ~3/100,000 in non-Finnish Europeans. The fact that this frequency is higher than the total frequency of LGMD caused by *CAPN3* variants and that homozygosity has only been reported in 2 patients but 5 unaffected individuals suggests that this variant is certainly not pathogenic alone in the heterozygous state and most likely also not in the homozygous state [14].

A major limitation of this study is the analysis of only one family, therefore not accounting for interfamilial variability. However, we think that literature analysis allowed at least in part to compensate for the shortcoming and therefore we think that the following conclusions concerning the *CAPN3* variants described in this article are warranted, without claiming absoluteness:

(I) The NM_000070.3: c.1746-20C>G, p.? variant alone is most likely not pathogenic neither in the homozygous nor in the heterozygous state.
(II) The NM_000070.3: c.1746-20C>G, p.? variant together with another pathogenic variant is pathogenic. Except for the variants listed in Supp. Tab. 2, it remains unknown which other second variants confer pathogenicity.

(I) and (II) are in keeping with the hypothesis by Mroczek et al. that c.1746-20C>G is a hypomorphic variant [14].

(III) The NM_000070.3: c.700G>A, p.Gly234Arg is pathogenic leading (a) in the heterozygous state most likely to “mild” phenotypes predominantly characterized by myalgia sometimes without overt paresis and sometimes associated with mild pareses (at least up to the age of ~60 years) and (b) in the compound heterozygous state together with c.1746-20C>G to a more severe phenotype characterized by progressive proximal pareses starting in the shoulder girdle progressing to pelvic and proximal leg muscles and often resulting in loss of full ambulation around the age of ~60 years. A caveat regarding the phenotype of heterozygous carriers lies in the fact that our patients suffered “only” from myalgia which is rather common. However, the severity of the myalgia leading to early retirement is very uncommon and no other family members experienced myalgias. Therefore, we firmly believe that the p.Gly234Arg variant is causative.

### Supplementary Information

Below is the link to the electronic supplementary material.

Supplementary file1 (10.6 KB)

Supplementary file2 (15.3 KB)
